# Combined hepatocellular-cholangiocarcinoma: which preoperative clinical data and conventional MRI characteristics have value for the prediction of microvascular invasion and clinical significance?

**DOI:** 10.1007/s00330-020-06861-2

**Published:** 2020-05-08

**Authors:** Xiaolong Wang, Wentao Wang, Xijuan Ma, Xin Lu, Shaodong Li, Mengsu Zeng, Kai Xu, Chun Yang

**Affiliations:** 1 Department of Radiology, Zhongshan Hospital, Fudan University, No. 180 Fenglin Road, Xuhui District, Shanghai, China; 2 Department of Radiology, The Affiliated Hospital of Xuzhou Medical University, No. 99 Huaihai West Road, Xuzhou, Jiangsu Province China; 3 School of Medical Imaging, Xuzhou Medical University, Xuzhou, Jiangsu Province China; 4 Department of Radiology, Xuzhou Central Hospital, Xuzhou, Jiangsu Province China

**Keywords:** Liver neoplasms, Neoplasm invasiveness, Magnetic resonance imaging

## Abstract

**Objectives:**

To explore which preoperative clinical data and conventional MRI findings may indicate microvascular invasion (MVI) of combined hepatocellular-cholangiocarcinoma (cHCC-CCA) and have clinical significance.

**Methods:**

The study enrolled 113 patients with histopathologically confirmed cHCC-CCA (MVI-positive group [*n* = 56], MVI-negative group [*n* = 57]). Two radiologists retrospectively assessed the preoperative MRI features (qualitative analysis of morphology and dynamic enhancement features), and each lesion was assigned according to the LI-RADS. Preoperative clinical data were also evaluated. Logistic regression analyses were used to assess the relative value of these parameters as potential predictors of MVI. Recurrence-free survival (RFS) rates after hepatectomy in the two groups were estimated using Kaplan–Meier survival curves and compared using the log-rank test.

**Results:**

The majority of cHCC-CCAs were categorized as LR-M. On multivariate analysis, a higher serum AFP level (OR, 0.523; 95% CI, 0.282–0.971; *p* = 0.040), intratumoral fat deposition (OR, 14.368; 95% CI, 2.749–75.098; *p* = 0.002), and irregular arterial peritumoral enhancement (OR, 0.322; 95% CI, 0.164–0.631; *p* = 0.001) were independent variables associated with the MVI of cHCC-CCA. After hepatectomy, patients with MVI of cHCC-CCA showed earlier recurrence than those without MVI (hazard ratio [HR], 0.402; 95% CI, 0.189–0.854, *p* = 0.013).

**Conclusion:**

A higher serum AFP level and irregular arterial peritumoral enhancement are potential predictive biomarkers for the MVI of cHCC-CCA, while intratumoral fat detected on MRI suggests a low risk of MVI. Furthermore, cHCC-CCAs with MVI may have worse surgical outcomes with regard to early recurrence than those without MVI.

**Key Points:**

*• Higher serum levels of AFP combined with irregular arterial peritumoral enhancement are independent risk factors for the MVI of cHCC-CCA, while fat deposition might be a protective factor.*

*• cHCC-CCA with MVI may have a higher risk of early recurrence after surgery.*

*• Most cHCC-CCAs were categorized as LR-M in this study, and no significant difference was found in MVI based on LI-RADS category.*

## Introduction

Combined hepatocellular-cholangiocarcinoma (cHCC-CCA) is a relatively uncommon subtype of primary hepatic malignant tumors, accounting for 2–5% of primary liver carcinomas (PLCs) [1–3]. Some studies have shown that cHCC-CCA has a biological behavior and prognosis that are intermediate between those of hepatocellular carcinoma (HCC) and intrahepatic cholangiocarcinoma (ICC) [4]; however, some reports have also stated that cHCC-CCA has a significantly worse prognosis than HCC and ICC, even after curative resection [5]. At present, the risk factors identified as being related to prognosis of cHCC-CCA are not uniform across studies because of the relatively low incidence and variations in sample size. Currently, studies have indicated that vascular invasion, lymph node metastasis, satellite nodules, and tumor size are major predictive factors for the prognosis of cHCC-CCA [6–8]. Studies have also shown that the level of cancer antigen 19-9 (CA19-9) or the presence of cirrhosis is a factor affecting the prognosis of cHCC-CCA [9, 10]. Scholars have not yet come to a consensus regarding the prognostic factors of cHCC-CCA. Although previous studies have confirmed that microvascular invasion (MVI) is a prognostic factor for tumor recurrence and is associated with poor survival outcomes in HCC [11–14] and ICC [15, 16], the relationship between prognosis and the presence of MVI in cHCC-CCA patients has not yet been established.

Currently, multiple magnetic resonance imaging (MRI) techniques have been used to improve the preoperative prediction of MVI in HCC [17–21]. Some imaging findings, such as “arterial peritumoral enhancement,” “tumor margin,” and “peritumoral hypointensity on hepatobiliary phase (HBP),” have been reported to be related to MVI in HCC [20]; some studies have shown that “incomplete tumor capsule” has a significant relationship with MVI in HCC [21]. A small number of studies have also used MRI to predict the MVI of mass-forming intrahepatic cholangiocarcinoma [22]. Currently, almost all the existing MRI studies have only described the imaging features or clinical characteristics of cHCC-CCA compared to those of pure HCC and ICC, usually with a small sample size [23–29]. Recently, studies have utilized LR-M features (including rim arterial phase hyperenhancement (APHE), peripheral “washout” appearance and delayed central enhancement) defined in version 2017 of the Liver Imaging Reporting and Data System (LI-RADS) to identify cHCC-CCA and HCC, and shown that LI-RADS categorization may provide prognostic information on cHCC-CCAs after surgery [30, 31]. However, these studies did not attempt to identify valuable preoperative MRI features indicating MVI in cHCC-CCA patients. Therefore, the purpose of this study was to evaluate the value of preoperative clinical data and conventional MRI findings including morphology, enhanced features, and the LI-RADS category for the preoperative prediction of the MVI of cHCC-CCA. Furthermore, the effect of MVI risk on the early recurrence of cHCC-CCA after surgery was estimated by the follow-up recurrence-free survival (RFS).

## Materials and methods

### Patient selection

**Fig. 1:**
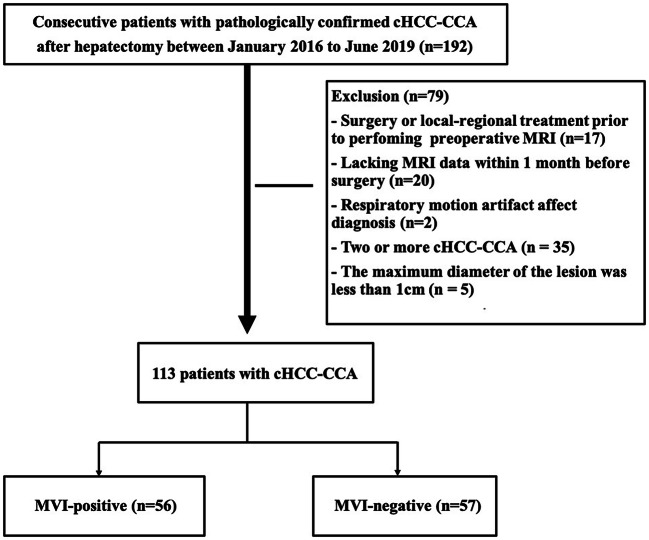
Flowchart detailing the patient selection process and exclusion criteria. In total, 113 patients with cHCC-CCA were enrolled in the final analysis. cHCC-CCA, combined hepatocellular-cholangiocarcinoma; MRI, magnetic resonance imaging; MVI, microvascular invasion

This retrospective study was approved by our institutional review board, and the need for informed patient consent was waived. Between January 2016 and June 2019, in total, 192 consecutive patients were confirmed by postoperative pathology to have cHCC-CCA and without extrahepatic metastasis by preoperative examinations. The inclusion criteria were as follows: (a) primary liver lesions without any prior treatment; (b) the MRI examinations were performed within 30 days before hepatectomy, and the MRI scans satisfied the diagnostic criteria; (c) there was a single mass without intrahepatic metastasis or lesions with multiple origins; and (d) the maximum diameter of the lesion was ≥ 1 cm. Finally, 79 cases were excluded for the following reasons: previous treatment history (*n* = 17, 8 cases of hepatectomy and 9 cases of transarterial chemoembolization [TACE] therapy); no MRI scans within 1 month before surgery (*n* = 20); poor MRI quality, including respiratory motion artifact effects (*n* = 2); two or more lesions of cHCC-CCA in the same liver (*n* = 35); and the maximum diameter of the lesion was less than 1 cm (*n* = 5). Finally, 113 patients with cHCC-CCA were enrolled in this study (Fig. 1).

### MRI acquisition

All patients were examined with a 24-channel 1.5-T magnetic scanner (uMR 560; United Imaging Healthcare). Routine plain-scan liver protocols consisted of a transverse T2-weighted breath-hold fat-suppressed fast spin-echo sequence, T1-weighted breath-hold in-phase and opposed-phase gradient echo sequence, and free-breath diffusion-weighted imaging (DWI) with a transverse single-shot spin-echo planar sequence (*b* value, 0, 50, and 500 s/mm^2^). Dynamic imaging was performed with a breath-hold T1-weighted 3-dimensional fat-suppressed quick spoiled gradient echo sequence before the intravenous administration of gadolinium-diethylenetriamine pentaacetic acid (Gd-DTPA) (Magnevist; Bayer HealthCare). Gd-DTPA was administered at a dose of 0.1 mmol/kg at a rate of 2 ml/s, followed by a 20-ml saline flush using a power injector (Spectris; Medrad). The arterial phase acquisition was triggered automatically by monitoring when the contrast media reached the ascending aorta. For subsequent acquisitions, dynamic T1-weighted MRI at 70–90 s (the portal venous phase) and 160–180 s (the delay phase) was performed. The detailed parameters of each acquisition sequence are shown in Table 1.

**Table 1:** Parameters of T1-weighted imaging, T2-weighted imaging and diffusion-weighted imaging

Parameter	FS-T2-weighted	T1-weighted IP and OP imaging	FS-T1-weighted quick3D BH	DWI
Repetition time (ms)	2693	115.8	4.4	2807
Echo time (ms)	85.6	4.7 and 2.2	2.2	75.7
Matrix size	201 × 288	230 × 288	192 × 256	115 × 128
Field of view (mm^2^)	380 × 360	400 × 280	400 × 280	380 × 300
Slice thickness (mm)	6.0	6.0	3.0	6.0
Slice gap (mm)	1.2	1.2	0	1.2

*FS* fat-suppression, *IP* in-phase, *OP* opposed-phase, *3D* three dimensional, *BH* breath-hold, *DWI* diffusion-weighted imaging

### Imaging analysis

All MRI scans were retrospectively analyzed together using a picture archiving and communication system (PACS; Pathspeed, GE Medical Systems Integrated Imaging Solutions) by two radiologists (X.L.W. and C.Y., with 7 and 13 years of experience in abdominal imaging, respectively). Both radiologists were aware that all patients had cHCC-CCA but were blinded to other clinical data, laboratory tests, and pathology results. A third experienced abdominal radiologist (K.X.) with more than 30 years of experience was invited to resolve any disagreements between the two observers.

### Qualitative analysis

The following qualitative imaging parameters of the lesions were evaluated on the plain scan: (a) shape of the tumors (globular, lobulated or irregular); (b) contour (smooth or nonsmooth margin); (c) homogeneous or heterogeneous on T2WI; (d) tumor location (right, left, both, or other liver lobe); (e) hemorrhage/hemosiderin; (f) intratumoral fat deposition; (g) necrosis; (h) upper abdominal lymphadenopathy (lymph nodes > 1 cm on the short axis); (i) peritumoral bile duct dilatation; and (j) hepatic capsular retraction. Dynamic enhancement characteristics were as follows: (A) arterial phase—(a) hypervascularity or nonhypervascularity; (b) homogeneity or heterogeneity enhancement; and (c) peritumoral enhancement patterns (assessed as detectable enhancing portion adjacent to the tumor border [wedge shaped], an extensive enhancement surrounding the tumor border [irregular shaped], or absent); (B) portal venous phase—(d) washout (nonperipheral washout or peripheral washout) and (e) enhancing capsule (complete, incomplete, or absent); (C) in the targetoid mass—(f) rim-APHE; (g) peripheral washout; (h) progressive central enhancement; and (i) targetoid diffusion restriction. In addition, all the lesions were categorized based on the LI-RADS v2018, LR-M (definitely or probably malignant, not HCC specific, including rim APHE, peripheral washout, and delayed and progressive concentric enhancement). Threshold growth was excluded because many patients had only one preoperative MRI examination.

### Clinical data and MVI pathological evaluation

The following clinical data were collected from the medical records: (a) demographic characteristics (age, sex); (b) etiology (hepatitis B or C virus infection, schistosomiasis, average daily alcohol consumption > 100 g/day, without obvious causes); (c) largest tumor diameter (divided into the 1–5 cm group and the > 5 cm group); (d) liver functional parameters (alanine aminotransferase [ALT], aspartate aminotransaminase [AST], γ-glutamyltranspeptidase [GGT], albumin [ALB], total bilirubin [TB], and direct bilirubin [DB]; and (e) tumor biomarkers (α-fetoprotein [AFP], carcinoembryonic antigen [CEA], and cancer antigen 19-9 [CA19-9]).

The pathological characteristics of the hepatectomy specimens were evaluated by a team of experienced pathologists (each individual had more than 12 years of experience in reading histopathological slices), who were blinded to the MRI and clinical results. MVI was defined as tumor cells within a vascular space lined by endothelium located in the periphery of the tumor at the tumor and liver parenchyma interface that was visible only by microscopy. The enrolled patients were divided into two groups (MVI-positive and MVI-negative) based on pathological characteristics.

### Follow-up RFS after surgery

All of the enrolled 113 patients with cHCC-CCAs underwent R0 liver resection (no residual tumor) within 30 days after the first MRI examination, with the surgical techniques and perioperative management the same as in previous reports [4]. Follow-up for RFS consisted of chest radiography, laboratory tests including serum AFP or protein induced by vitamin k absence or antagonist-II (PIVKA-II), and abdominal MRI at 1 month after surgery; if there was no recurrence, the patient was reexamined every 2–3 months. If only the level of a tumor marker increased without any radiographic evidence of a new tumor, follow-up was continuous until a tumor presented on imaging, at which point the time of recurrence was recorded.

### Statistical analysis

All statistical analyses were performed using SPSS 20.0 and MedCalc software (version 15.0). Normally distributed data are expressed as the means ± standard deviations, and comparisons between the two groups were performed using independent sample *t* tests. The data with skewed distributions are expressed as the medians (25%, 75%), and comparisons between the two groups were performed using rank sum tests. Categorical variables are reported as the numbers of cases and percentages, and *χ*^2^ or Fisher’s exact tests were used. Comparisons between groups of categorical variables were performed by one-way analysis of variance. Parameters were analyzed using univariate and multivariate logistic regression to determine whether they were independent risk factors predicting MVI (the univariate analysis was performed first, and only those parameters found to have statistical significance were used in the stepwise multivariate logistic regression). A *p* value less than 0.05 indicated a significant difference. The odds ratio (OR) and 95% confidence interval (CI) were calculated. The sensitivity, specificity, accuracy, positive predictive value (PPV), and negative predictive value (NPV) were calculated for each significant finding and combinations of significant findings on multivariate logistic regression with regard to predicting MVI. The RFS after hepatectomy in two groups were estimated using Kaplan–Meier survival curves and compared using the log-rank test.

## Results

### Patient clinical and MR characteristics

The comparisons of patient clinical characteristics stratified by the MVI status and data are detailed in Table 2. The results revealed MVI-positive lesions in 56 patients (49.6%) and MVI–negative lesions in 57 patients (50.4%). There were significant differences in the tumor size > 5 cm and the level of serum AFP ≥ 400 ng/ml between MVI-positive and MVI-negative groups (*p* = 0.006 and *p* = 0.022, respectively), but when the serum level of AFP was between 20 and 400 ng/ml, there was no significant differences between the two groups. No significant differences (*p* > 0.05) were found in age, sex, etiology, liver functional parameters, or the levels of CA19-9 and CEA between the two groups (Table 2).

**Table 2:** Clinical characteristics of cHCC-CCA according to MVI

Clinical parameters	MVI-positive (*n* = 56)	MVI-negative (*n* = 57)	*p* value
Age (years) ^a^	56.9 ± 11.4	52.7 ± 11.6	0.0561
Sex (male:female)	37:19	40:17	0.640
Largest diameter (cm)^a^	5.4 ± 3.2.	3.7 ± 1.9	0.0009
1–5 cm	26 (46.4)	41 (71.9)	0.006
> 5 cm	30 (53.6)	16 (28.1)	
Etiology^†^			0.290
Hepatitis B virus	42 (75.0)	48 (84.2)	
Hepatitis C virus	1 (1.8)	0 (0)	
None or other	13 (23.2)	9 (15.8)	
Liver functional parameters			
Total bilirubin > 20 (μmol/L)	8 (14.3)	9 (15.8)	0.823
Direct bilirubin > 7 (μmol/L)	7 (12.5)	12 (21.1)	0.224
Alanine aminotransferase > 40 (IU/L)	15 (26.8)	18 (31.6)	0.575
Aspartate aminotransferase > 40 (IU/L)	13 (23.2)	10 (17.5)	0.454
γ-Glutamyltranspeptidase > 60 (IU/L)	26 (46.4)	28 (49.1)	0.774
Albumin < 35 (g/L)^†^	3 (5.4)	2 (3.5)	0.679
Tumor markers			
Alpha-fetoprotein ≥ 20 and < 400 (ng/ml)	21 (37.5)	22 (38.6)	0.796
Alpha-fetoprotein ≥ 400 (ng/ml)	18 (32.1)	8 (14.0)	0.022
Cancer antigen 19-9 > 37 (U/ml)	13 (23.2)	14 (24.6)	0.867
Carcinoembryonic antigen > 5 (ng/ml)	12 (21.4)	8 (14.0)	0.303

Data are numbers of patients (percentage), unless otherwise specified

^a^Data are means ± standard deviations

^†^Data were compared using the Fisher’s exact test. The ages were compared using an independent sample *t* test. Excepted where indicated, data were compared using the *χ*^2^ test

**Fig. 2:**
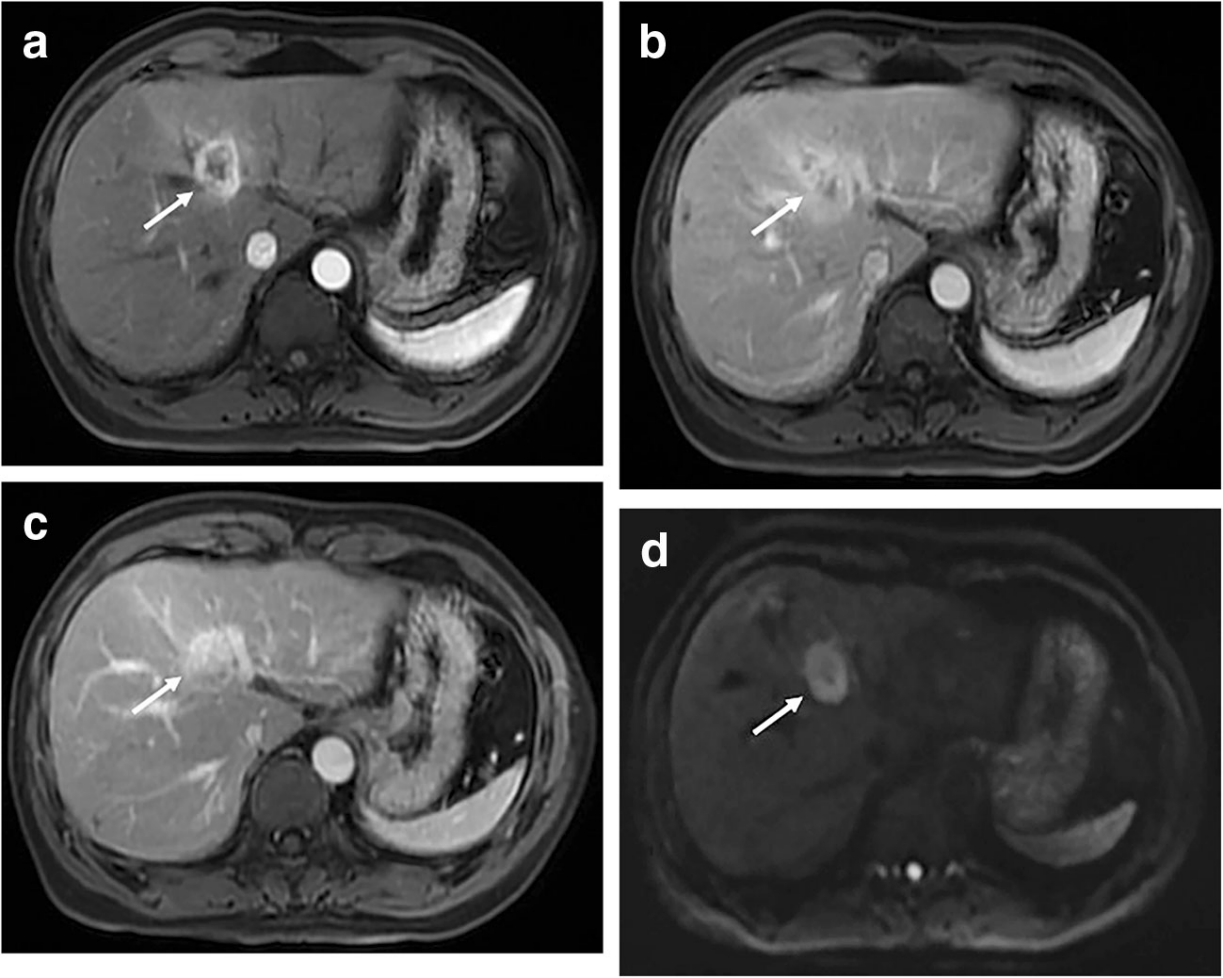
Images in a 57-year-old man with cHCC-CCA categorized as LR-M with MVI. **a** Axial arterial phase image shows a 3.5-cm rim hyperenhancement lesion (arrow) in segment IV of the liver. **b** Portal venous phase image shows continuous peripheral enhancement and progressive central enhancement (arrow). **c** Delay phase image shows a further progressive central enhancement appearance (arrow). **d** Diffusion-weighted image shows targetoid appearance (*b* = 500 s/mm^2^) with peripheral hyperintensity and central relatively hypointensity (arrow)

Among the recorded MRI characteristics (Table 3), tumor shape (*p* = 0.025), hemorrhage/hemosiderin (*p* = 0.032), intratumoral fat deposition (*p* = 0.013), upper abdominal lymphadenopathy (*p* = 0.010), the arterial phase peritumoral enhancement pattern (*p* < 0.001), and peritumoral bile duct dilatation (*p* = 0.044) were significantly associated with MVI. Most (89/113, 78.8%) of the cHCC-CCA could be properly categorized as LR-M (Fig. 2), and no significant difference in MVI was found based on the LI-RADS category (*p* = 0.819). Other features did not differ between the two groups (Table 3).

**Table 3:** Comparison of qualitative data obtained on MRI features stratified by MVI status

MRI features	MVI-positive (*n* = 56)	MVI-negative (n = 57)	*p* value
Shape^†^			0.025
Irregular	7 (12.5)	3 (5.3)	
Lobulated	38 (67.8)	30 (52.6)	
Globular	11 (19.6)	24 (42.1)	
Contour smooth	14 (25.0)	20 (35.1)	0.242
Homogeneity T2	34 (60.1)	39 (68.4)	0.392
Hemorrhage / hemosiderin	16 (28.6)	7 (12.3)	0.032
Fat deposition^†^	3 (5.4)	13 (22.8)	0.013
Necrosis	20 (35.7)	14 (24.6)	0.196
Upper abdominal lymphadenopathy	22 (39.3)	10 (17.5)	0.010
Location^†^			0.953
Right liver lobe	40 (71.4)	41 (71.9)	
Left live lobe	13 (23.2)	12 (21.1)	
Caudate lobe or border area	3 (5.4)	4 (7.0)	
Arterial phase hyperenhancement	50 (89.2)	56 (98.2)	0.061
Arterial phase homogeneity enhancement^†^	2 (3.6)	9 (15.8)	0.053
Arterial phase peritumoral enhancement			< 0.001
Absent	13 (23.2)	39 (69.4)	
Wedge shaped	19 (33.9)	9 (15.8)	
Irregular	24 (42.9)	9 (15.8)	
Washout at portal venous phase	28 (50.0)	30 (52.6)	0.780
Enhancing capsule			
Complete	11 (19.6)	14 (24.6)	0.275
Incomplete	12 (21.4)	6 (10.5)	
Absent	33 (58.9)	37 (64.9)	
Targetoid mass			
Rim arterial phase hyperenhancement	27 (48.2)	33 (57.9)	0.303
Peripheral washout	12 (21.4)	18 (31.6)	0.222
Progressive central enhancement	38 (67.8)	43 (75.4)	0.371
Targetoid diffusion restriction^†^	1 (1.8)	5 (8.8)	0.206
Peritumoral bile duct dilatation	14 (25.0)	6 (10.5)	0.044
Surface retraction^†^	1 (1.8)	1 (1.8)	1.000
LI-RADS categorization^†^			0.819
LR-4	1 (1.8)	1 (1.8)	
LR-5	12 (21.4)	10 (17.5)	
LR-M	43 (76.8)	46 (80.7)	
LR-TIV^†^	7 (12.5)	2 (3.5)	0.124

The data are presented as the number (%) of patients

†Data were compared using the Fisher’s exact test. LR-4 probably HCC, LR-5 definitely HCC, LR-M definitely or probably malignant, not HCC specific, LR-TIV tumor in vein

### Univariate and multivariate analyses

Univariate logistic regression analysis showed that there were eight risk factors that were significantly related to the MVI of cHCC-CCA (Table 4). A larger tumor size (*p* = 0.002), a higher serum level of AFP (*p* = 0.013), an irregular shape (*p* = 0.005), hemorrhage/hemosiderin (*p* = 0.036), intratumoral fat deposition (*p* = 0.014), upper abdominal lymphadenopathy (*p* = 0.012), arterial phase homogeneity enhancement (*p* = 0.044), and irregular arterial peritumoral enhancement (*p* < 0.001) were associated with MVI. These parameters were analyzed using multivariate logistic regression. Higher serum levels of AFP (odds ratio [OR], 0.523; 95% confidence interval [CI], 0.282–0.971; *p* = 0.040), intratumoral fat deposition (OR, 14.368; 95% CI, 2.749–75.098; *p* = 0.002), and irregular arterial peritumoral enhancement (OR, 0.322; 95% CI, 0.164–0.631; *p* = 0.001) were independent variables associated with the MVI of cHCC-CCA.

**Table 4:** Univariate and multivariate analyses of risk factors for the MVI of cHCC-CCA

Risk factor	Univariate analysis		Multivariate analysis	
	Odds ratio (95% CI)	*p* value	Odds ratio (95% CI)	*p* value
Age (years)^a^	0.968 (0.936–1.001)	0.060	…	…
Sex (male:female)	0.828 (0.375–1.828)	0.640	…	…
Largest diameter (cm)	0.772 (0.655–0.908)	0.002	1.010 (0.788–1.94)	0.937
Alpha-fetoprotein ≥ 400 (ng/ml)	0.533 (0.324–0.876)	0.013	0.523 (0.282–0.971)	0.040
Cancer antigen 19-9 > 37 (U/ml)	1.077 (0.453–2.558)	0.060	…	…
Carcinoembryonic antigen > 5 (ng/ml)	0.490 (0.187–1.282)	0.146	…	…
Shape	…	…	…	…
Irregular	0.387 (0.199–0.753)	0.005	0.718 (0.293–1.758)	0.469
Lobulated*	…	…	…	…
Globular*	…	…	…	…
Contour smooth	1.621 (0.719–3.658)	0.244	…	…
Hemorrhage/hemosiderin	0.350 (0.131–0.933)	0.036	0.910 (0.252–3.280)	0.885
Fat deposition	5.220 (1.398–19.490)	0.014	14.368 (2.749–75.098)	0.002
Necrosis	0.972 (0.453–2.085)	0.942	…	…
Upper abdominal lymphadenopathy	0.328 (0.138–0.783)	0.012	0.358 (0.118–1.087)	0.070
Arterial phase homogeneity enhancement	5.062 (1.041–24–596)	0.044	1.932 (0.295–12.643)	0.492
Arterial phase peritumoral enhancement	…	…	…	…
Absent*	…	…	…	…
Wedge shaped*	…	…	…	…
Irregular	0.332 (0.201–0.550)	< 0.001	0.322 (0.164–0.631)	0.001
Enhancing capsule	0.675 (0.320–1.426)	0.303	…	…
Targetoid mass	…	…	…	…
Rim arterial phase hyperenhancement	1.477 (0.703–3.103)	0.303	…	…
Peripheral washout	1.692 (0.724–3.952)	0.224	…	…
Progressive central enhancement	1.455 (0.639–3.315)	0.373	…	…
Targetoid diffusion restriction	5.288 (0.598–46.794)	0.134	…	…
Peritumoral bile duct dilatation	0.353 (0.125–0.998)	0.053	…	…
Surface retraction	0.982 (0.060–16.097)	0.990	…	…
LR-M	1.205 (0.539–2.690)	0.650	…	…

^a^Data are the means ± standard deviations

*Data were used as the reference variable. LR-M definitely or probably malignant, not HCC specific

**Fig. 3:**
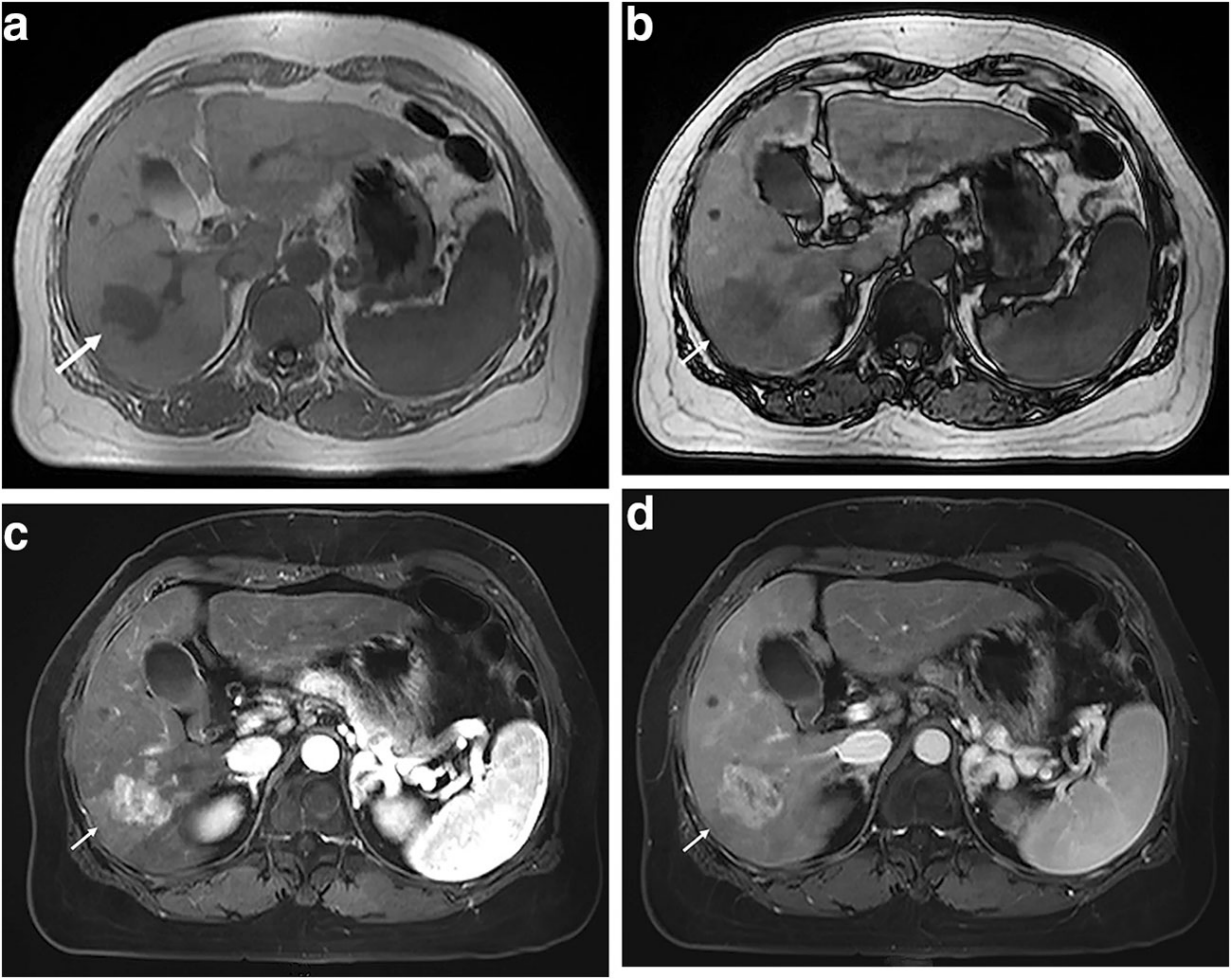
Images in a 58-year-old woman with cHCC-CCA with MVI; her serum level of AFP was 1885 ng/ml. **a** In-phase MR image shows a 3.0-cm hypointense irregular mass (arrow) in segment VI of the liver. **b** On the opposed-phase image, there was no obvious signal drop in the lesion (arrow), indicating the absence of an unambiguous fatty-containing lesion. **c** Axial arterial phase image shows a hypervascular mass with irregular peritumoral enhancement (arrow). **d** Portal venous phase image shows nonsmooth tumor margin and peritumoral slight hypointensity (arrow)

The sensitivity, specificity, accuracy, PPV, and NPV for the prediction of MVI by the three significant factors and their combination are shown in Table 5. When all three factors were combined (Fig. 3), the specificity was 98.2% (56/57), and the sensitivity was 12.5% (7/56).

**Table 5:** Diagnostic performance of independent risk factors for the prediction of MVI in cHCC-CCA

Factors	Sensitivity	Specificity	Accuracy	PPV	NPV
Alpha-fetoprotein ≥ 400 (ng/ml)^a^	32.1 (18/56)	86.0 (49/57)	59.3 (67/113)	69.2 (18/26)	56.3 (49/87)
Without intratumoral fat deposition^b^	94.6 (53/56)	22.8 (13/57)	58.4 (66/113)	54.6 (53/97)	81.3 (13/16)
Irregular peritumoral enhancement^c^	42.9 (24/56)	84.2 (48/57)	63.7 (72/113)	72.7 (24/33)	60.0 (48/80)
Combination of a and b	32.1 (18/56)	87.7 (50/57)	60.2 (68/113)	72.0 (18/25)	56.8 (50/88)
Combination of b and c	39.3 (22/56)	91.2 (52/57)	65.5 (74/113)	81.5 (22/27)	60.5 (52/86)
Combination of a and c	17.9 (10/56)	96.5 (55/57)	57.5 (65/113)	83.3 (10/12)	54.5 (55/101)
Combination of all three factors	12.5 (7/56)	98.2 (56/57)	55.8 (63/113)	87.5 (7/8)	53.3 (56/105)

Data are presented as percentages. Data in parentheses are the numbers of subjects used to calculate the percentage

^a^Alpha-fetoprotein ≥ 400 (ng/ml)

^b^Without intratumoral fat deposition

^c^Irregular peritumoral enhancement

*PPV* positive predictive value, *NPV* negative predictive value

### RFS outcomes after surgery

**Fig. 4:**
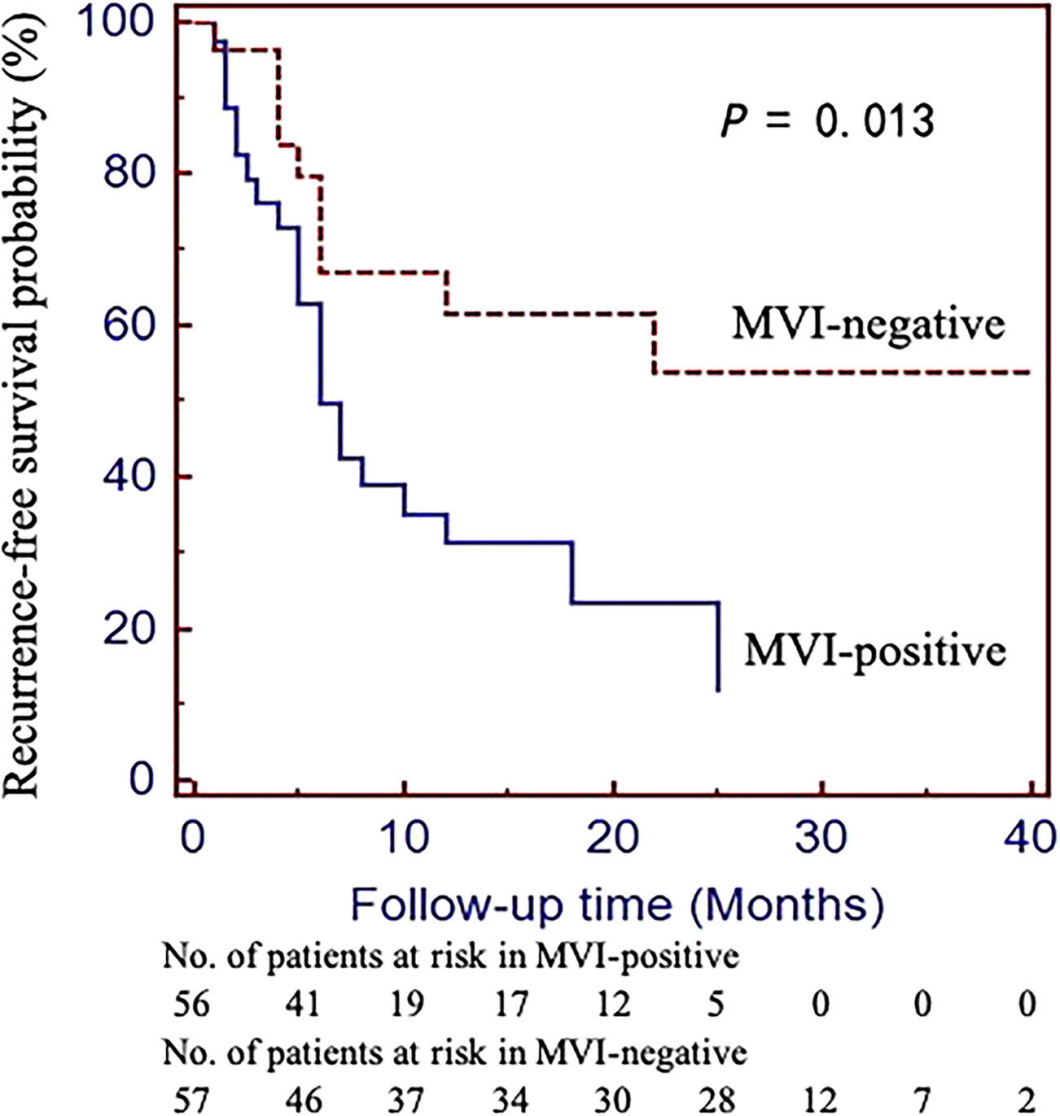
Kaplan–Meier survival curve for the recurrence-free survival of patients with cHCC-CCAs that were MVI-positive and MVI-negative (HR = 0.402; 95% CI, 0.189–0.854, *p* = 0.013). cHCC-CCA, combined hepatocellular-cholangiocarcinoma; MVI, microvascular invasion; HR, hazard ratio; CI, confidence interval

All 113 patients with cHCC-CCAs received R0 liver resection (no residual tumor) within 30 days after the first MRI examination. After hepatectomy, patients with MVI of cHCC-CCA had a median RFS of 10.8 months (range 1–25 months), while those without MVI had a median RFS of 25.4 months (range 1–40 months), and the early recurrence rates (< 2 years) were estimated to be 83.9% (47/56) and 49.1% (28/57), respectively. There was a significant difference in RFS between patients with MVI-positive and MVI-negative tumors (hazard ratio [HR], 0.402; 95% CI, 0.189–0.854, *p* = 0.013). Kaplan–Meier survival curves were generated (Fig. 4).

## Discussion

Our results illustrated that a higher serum level of AFP and irregular arterial phase peritumoral enhancement may indicate a higher risk of the MVI of cHCC-CCA, while intratumoral fat detected on MRI suggests a lower risk. Combining these three findings for the prediction of MVI resulted in specificity greater than 98%. In addition, cHCC-CCAs with MVI may have worse surgical outcomes with regard to early recurrence than those without MVI.

Previously, a few studies reported that a higher serum level of AFP was one of the independent risk factors associated with MVI in HCC [32, 33] and ICC [34] patients. Our findings also showed that a higher serum level of AFP was an independent predictor of MVI in cHCC-CCA, but when the serum level of AFP was between 20 and 400 ng/ml, there were no significant differences in MVI. Furthermore, some studies have suggested that the clinical characteristics of cHCC-CCA are similar to those of HCC; for example, the majority of cHCC-CCAs occur against a background of positive hepatitis B serology and cirrhosis, and the patients are predominately male [28, 35]. Our results were consistent with these studies. In this study, the patients had a sex ratio (male: female) of 77:36, and most patients (79.6%) had been infected with the hepatitis B virus; however, no significant differences in age, sex, or etiology were found regard to in MVI.

Intratumoral fat deposition was an additional significant factor for predicting a lower risk of the MVI of cHCC-CCA in our study, which was consistent with some reports. Min et al [36] described that intratumoral fat was one of the independent variables for suggesting a lower risk of the MVI of HCC. A few studies have suggested that intratumoral fatty changes are associated with favorable tumor grades on histologic examination and a lower likelihood of MVI; therefore, fat-containing lesions may predict a more favorable prognosis than non-fat-containing lesions [36, 37]. Moreover, as is well known, fatty changes in HCC are associated with ischemia, which may be related to a reduced normal portal vein blood supply [38]. Increased intratumoral fat may indicate less aggressive HCC, as evidenced by the fact that HCC with diffuse fat tends to grow slowly. Because our sample size for fat-containing cHCC-CCA with MVI was relatively small, the relationship between intratumoral fat and the prognosis of cHCC-CCA remains to be further studied.

Our study also showed that irregular arterial phase peritumoral enhancement was a significant MRI finding predicting the MVI of cHCC-CCA. Many reports [18, 20, 39] have shown that arterial peritumoral enhancement is an independent predictive factor of MVI in HCC. To date, few studies have described the relationship between peritumoral enhancement of cHCC-CCA and MVI. The mechanism of hemodynamic changes in this type of MRI feature is interpreted as a decrease in or disappearance of portal blood flow due to tumor thrombosis in the microportal branch around the tumor, resulting in compensatory hepatic arterial hyperperfusion [40]. In addition, although previous studies have reported that a large tumor size could be considered a major predictor of HCC with MVI [36], it has not always been considered an independent predictor of the MVI of HCC [18, 19]. In this study, tumor size, tumor shape, intratumoral hemorrhage, upper abdominal lymphadenopathy, and arterial phase heterogeneity enhancement were important risk factors for the MVI of cHCC-CCA in univariate analysis, but they were not independent factors predicting MVI.

It has been reported that MVI is one of the most important prognostic factor for the early recurrence of HCC after hepatic resection or radiofrequency ablation [20, 39]; we also found that cHCC-CCAs with MVI may have worse surgical outcomes with regard to early recurrence than those without MVI. Recent studies [30] have reported that patients with cHCC-CCAs in the LR-M category had a higher early recurrence rate (≤ 6 months) than those with cHCC-CCAs in the LR-5/4 categories. While there was no significant difference in RFS, cHCC-CCAs mimicking HCCs on imaging (LR-5/4) may have improved surgical outcomes. Unlike this study, a substantial proportion of cHCC-CCAs were categorized as LR-M (78.8%, 89/113) in our study; nevertheless, no significant difference in the MVI of cHCC-CCA was found based on the LI-RADS categories.

This study has several limitations. First, because this research was a single-center and retrospective study, there might have been selection bias. Second, tumor size and the number of lesions were confined to larger than 1 cm in maximum diameter and a single mass in this study; therefore, the conclusions cannot be generalized to other size lesions or two or more lesions. Third, the data for overall survival (OS) were not available; thus, the relationship between the MVI of cHCC-CCA and OS requires further research in the future. Fourth, in this study, Gd-DTPA was used as a contrast agent for MRI; therefore, further research is warranted on gadoxetic acid–enhanced MRI for the identification of the MVI of cHCC-CCA. Fifth, our sample size for fat-containing cHCC-CCA with MVI was relatively small which resulted in a low diagnostic sensitivity when all the three parameters were combined; therefore, more patients needed to be enrolled to clarify the diagnostic efficacy. Finally, in our study, cHCC-CCA was assessed only as either MVI-positive or MVI-negative. In a recent study [41], MVI was further categorized into different grades based on the number of vessels invaded. Further study is needed to assess the relationship between preoperative clinical or MRI findings and different grades of MVI of cHCC-CCA.

In summary, the proportion of MVI-positive patients accounts for approximately half of all cHCC-CCA patients. Higher serum levels of AFP and irregular arterial peritumoral enhancement were independent variables associated with the MVI of cHCC-CCA, while fat deposition might be a protective factor. In addition, cHCC-CCA with MVI may have a higher early recurrence rate after surgery.

## Abbreviations

AFP: Alpha-fetoprotein
APHE: Arterial phase hyperenhancement
CA19-9: Cancer antigen 19-9
CEA: Carcinoembryonic antigen
cHCC-CCA: Combined hepatocellular-cholangiocarcinoma
CI: Confidence interval
DWI: Diffusion-weighted imaging
Gd-DTPA: Gadopentate dimeglumine
HBP: Hepatobiliary phase
HCC: Hepatocellular carcinoma
ICC: Intrahepatic cholangiocarcinoma
LI-RADS: Liver Imaging Reporting and Data System
LR-M: Probably or definitely malignant, not HCC specific
MRI: Magnetic resonance imaging
MVI: Microvascular invasion
NPV: Negative predictive value
OR: Odds ratio
OS: Overall survival
PLCs: Primary liver carcinomas
PPV: Positive predictive value
RFS: Recurrence-free survival
TACE: Transarterial chemoembolization
